# Pain and Body Awareness: Evidence from Brain-Damaged Patients with Delusional Body Ownership

**DOI:** 10.3389/fnhum.2013.00298

**Published:** 2013-06-20

**Authors:** Lorenzo Pia, Francesca Garbarini, Carlotta Fossataro, Luca Fornia, Anna Berti

**Affiliations:** ^1^Psychology Department, University of Turin, Turin, Italy; ^2^Neuroscience Institute of Turin, University of Turin, Turin, Italy; ^3^Section of Physiology, Department of Neuroscience, University of Parma, Parma, Italy

**Keywords:** body ownership, disownership, pain, brain-damaged patients, body awareness

## Abstract

A crucial aspect for the cognitive neuroscience of pain is the interplay between pain perception and body awareness. Here we report a novel neuropsychological condition in which right brain-damaged patients displayed a selective monothematic delusion of body ownership. Specifically, when both their own and the co-experimenter’s left arms were present, these patients claimed that the latter belonged to them. We reasoned that this was an ideal condition to examine whether pain perception can be “referred” to an alien arm subjectively experienced as one’s own. Seventeen patients (11 with, 6 without the delusion), and 10 healthy controls were administered a nociceptive stimulation protocol to assess pain perception. In the OWN condition, participants placed their arms on a table in front of them. In the ALIEN condition, the co-experimenter’s left (or right) arm was placed alongside the participants’ left (or right) arm, respectively. In the OWN condition, left (or right) participants’ hand dorsum were stimulated. In the ALIEN condition, left (or right) co-experimenter’s hand dorsum was stimulated. Participants had to rate the perceived pain on a 0–5 Likert scale (0 = no pain, 5 = maximal imaginable pain). Results showed that healthy controls and patients without delusion gave scores higher than zero only when their own hands were stimulated. On the contrary, patients with delusion gave scores higher than zero both when their own hands (left or right) were stimulated and when the co-experimenter’s left hand was stimulated. Our results show that in pathological conditions, a body part of another person can become so deeply embedded in one’s own somatosensory representation to effect the subjective feeling of pain. More in general, our findings are in line with a growing number of evidence emphasizing the role of the special and unique perceptual status of body ownership in giving rise to the phenomenological experience of pain.

## Introduction

Pain perception is at the root of animal life and is vital to survival. Being able to perceive pain protects us by triggering a reflexive withdrawal from potentially dangerous stimuli before we can suffer further injury, it tells us that an injury is about to occur, it lets us know when we need to seek medical help, and teaches us what behaviors to avoid in the future.

Given such a higher evolutionary significance of pain perception, one would be keen to consider it an all-or-none phenomenon or, at least, tightly regulated by the input features (e.g., stimulus modality, intensity, duration, etc.). However, the current evidence on pain perception tells us a different story. The neural encoding of internal or external events that injures, or threatens to injure, our body is known as nociception. Nociceptors (i.e., pain receptors) detect when thermal, chemical, and mechanical stimuli are above a threshold. Then, the information is sent through the spinal cord and the brainstem up to the cortex. Nociception automatically triggers a variety of autonomic responses (e.g., hypertension, tachycardia, and fainting). Nonetheless, it can also generate an emotional and unpleasant subjective experience related to the stimulation known as pain perception.

It is known that the relationship between noxious stimuli (input) and its pain perception (output) is usually non-linear. Along the route from nociception to pain, several psychological, and/or cognitive factors modulate the physiology of pain before it becomes part of our consciousness. It is known, for instance, that pain perception can be ameliorated by the context as demonstrated by the fact that soldiers suffering from compound fractures during battles can report only twinges of pain (Horstman and Flax, 1999). The same has been reported with respect to the focus of attention: noxious stimuli are perceived less intense when people are distracted by other potentially relevant stimuli (Terkelsen et al., 2004). In addition, expectations have a crucial role, as shown by the fact that healing expectations can enhance the placebo effect (Turner et al., 1994). In some cases, a person can even experience pain without nociception. Amputees, for instance, can experience phantom pain that is painful perception referred to the absent limb (Ramachandran and Hirstein, 1998).

Due to its complexity, pain perception does not rely on the activity of a single brain structure but, rather, on a large distributed cortical/subcortical network known as pain matrix (see, for instance, Iannetti and Mouraux, 2010). According to the projections sites from either the medial or the lateral thalamic structures to the cortex, this system can be broadly subdivided in two subcomponents a “medial pain system” that processes the emotional aspects (e.g., unpleasantness) and a “lateral pain system” that subserves intensity, location, and duration (Albe-Fessard et al., 1985). The first system includes amygdala, anterior cingulate cortex, hippocampus, hypothalamus, locus coeruleus, and periaqueductal gray matter, whereas the second involves primary and secondary somatosensory cortex, parietal operculum, and insula. However, the crucial problem is that the extent to which this activity represents, or even correlates, with pain perception is unclear since those brain responses can be generated in non-nociceptive conditions (e.g., Craig et al., 1996).

Another interesting point related to pain perception is its connection with body ownership, which is the conscious experience that bodily states are so clearly and inexorably “mine” (Gallagher, 2000). Experiencing the body as one’s own is a prerequisite for almost every cognitive function, it is intimately related to human’s self-consciousness, and it shapes individual psychological identity. Indeed, our body constantly receives flows of inputs (i.e., touch, vision, proprioception, and interception). Notwithstanding, in order to be considered as potentially noxious (i.e., relevant) stimuli, these inputs must be invariably perceived as parts of one’s own body and as unique to oneself. Put in another way, human’s experience of pain is strictly dependent from the way we represent the body itself and from the sense that it is *my* body that is undergoing a certain experience (i.e., body ownership).

A first hint with respect to the relationship between body ownership and pain is the feeling of “foreignness” toward the affected body part often observed in patients affected by regional pain syndrome (Bultitude and Rafal, 2010). Perhaps, the more compelling evidence of the tight link between body ownership and pain has been obtained in healthy participants by means of an experimental manipulation in which the physical constraints subserving body ownership are altered. Such a paradigm, known as the “rubber hand illusion” (Botvinick and Cohen, 1998), shows that synchronous touches onto a visible rubber hand and onto the hidden participants’ hand produce the compelling feeling of ownership of that hand (e.g., Botvinick and Cohen, 1998; Farnè et al., 2000; Ehrsson et al., 2004; Tsakiris and Haggard, 2005; Costantini and Haggard, 2007; Longo et al., 2008). This is demonstrated both subjectively (i.e., by a self-report questionnaire) and behaviorally (i.e., the location of one’s own hand is shifted toward the rubber hand).

Crucially, recent studies (Capelari et al., 2009; Mohan et al., 2012) showed that the rubber hand illusion arises also with synchronous tactile noxious stimuli (but, see also Valenzuela-Moguillansky et al., 2011) and the effects do not differ from those obtained with non-noxious tactile stimuli (Capelari et al., 2009). These experiments suggest that pain can be referred to the rubber hand as long as it is being perceived as part of one’s own body (Capelari et al., 2009; Mohan et al., 2012). More in general, they indicate that the neurocognitive mechanisms involved in localizing touch during the illusion might be, at least in part, similar to those required to localize pain. This is an important point since it is often assumed that touch can be referred to external objects (e.g., tips of tools; Iriki et al., 1996), whereas pain cannot.

The effect observed during the rubber hand illusion implies that whenever we feel an external body part as part of our’s own body, noxious, exactly as non-noxious, stimuli can be potentially referred to it. During the illusion, painful perception is reported to arise from the rubber hand while one’s own hand is actually receiving the stimulation. In the present paper, we asked a further question that is whether an altered feeling of body ownership can affect painful perception to a degree that it is possible to experience the pain delivered to an alien hand without any simultaneous stimulation on one’s own hand.

We aimed at answer this question within a neuropsychological approach. Indeed, patients’ counterintuitive behavior can potentially unmask the inadequacies of theories on human brain functioning hidden from the view in the intact brain (see Churchland, 1986 for a discussion on this point). In the present context, studying the abnormalities of the integration among the different components of body ownership due to brain damages has a key role in addressing questions regarding the structure and functional signature of body consciousness. Here, we focused on a subgroup of right brain-damaged patients affected by a selective disturbance of body ownership, which is they misattribute another person’s arm to themselves (Garbarini et al., 2013). Specifically, when both their own and the co-experimenter’s left arms were visible, they tended to claim that the latter belonged to them. Moreover, these patients treated and cared the co-experimenter’s left arm as their own’s one even if provided with contrary evidence coming from different sensory modalities. Hence, we compared patients with such a delusion with participants who did not have this experience (i.e., right brain-damaged patients without the delusion and healthy subjects). The task required participants to rate the perceived pain evoked by nociceptive stimulators administered under different conditions (i.e., stimulation of both the participant and the co-experimenter’s hands). If conscious experience of owning an alien arm is the result of a profound embodiment of the alien arm into the participant’s sensory-motor circuits, it should produce a pain perception when stimuli are applied onto the co-experimenter’s left hand only in patients affected by the delusion.

## Materials and Methods

### Baseline assessment

Seventeen consecutive right-handed patients (five women; mean age 65.93 years, SD = 12.89 years; mean educational level 9.43 years, SD = 5.31 years) with right hemisphere lesion and 10 age and educational level-matched right-handed healthy subjects participated in the study after having given written informed consent according to the declaration of Helsinki. Patients’ demographic, clinical, and neuropsychological data are reported in Table 1. Patients were admitted to a rehabilitation center for the treatment of their neurocognitive deficits and none of them had a history of substance abuse or previous neurological diseases. All suffered from a single right hemisphere lesion confirmed by CT or MRI scans. Lesions involved several cortical/subcortical structures, as well as white matter, within fronto-temporo-parietal regions. Patients were initially screened with the mini mental state examination (Measso et al., 1993) to exclude the presence of severe cognitive impairments. Contralesional somatosensory, motor, visual field defects as well as unawareness for motor and somatosensory deficits were assessed according to the a standard neurological exam (see Pia et al., 2013 for details). It is worth noticing that somatosensory defects were assessed with both tactile and noxious (pinprick stimulators) stimuli. No dissociation was found between presence/absence of the defects, as well as unawareness of them. In other words, administering non-noxious or noxious stimuli did not make any difference. The presence of left extrapersonal neglect was assessed with the behavioral and conventional scales of the Behavioral Inattention Test (Wilson et al., 1987), and left personal neglect with the Fluff test (Cocchini et al., 2001). Patients were also evaluated for somatoparaphrenia (Fotopoulou et al., 2011) and verbal asomatognosia (Feinberg et al., 1990).

**Table 1:** **Demographic and clinical data of patients**.

Id	G	S	A	S	E	D	NE			A		MMSE	Neglect			Som	Aso	Mis			
							V	M	S	M	S		EP		P			Arm		Arms	
													BIT-C	BIT-B	Fluff			Pt	Co-ex	Pt	Co-ex
1	E+	F	72	5	I	60	0–0	3–3	3–3	0–0	2–2	28	66	60	0	N	N	100	100	0	100
2	E+	F	50	18	I	40	0–0	3–3	3–3	0–0	2–2	29	139	79	0	N	N	100	100	50	50
3	E+	M	78	8	I	60	0–0	0–0	3–3	0–0	2–2	29	50	42	0	N	N	100	100	0	100
4	E+	M	82	8	I	45	0–0	3–3	2–2	0–0	2–2	27	89	46	0	N	N	100	100	0	100
5	E+	F	75	5	I	40	0–0	3–3	2–2	2–2	2–2	28	90	59	0	N	N	100	100	0	100
6	E+	M	68	5	I	70	1–1	3–3	3–3	0–0	2–2	25	14	1	3	N	N	100	100	0	100
7	E+	M	64	17	I	50	1–1	3–3	3–3	0–0	2–2	25	135	40	2	N	N	100	100	0	100
8	E+	F	77	17	H	35	0–0	3–3	3–3	0–0	0–0	28	140	73	0	N	N	100	100	0	100
9	E+	M	55	5	I	30	0–0	3–3	2–2	0–0	0–0	18	17	8	3	Y	N	100	100	0	100
10	E+	M	69	8	I	30	0–0	3–3	0–0	0–0	0–0	27	138	75	0	N	N	100	100	0	100
11	E+	M	64	17	I	50	1–1	3–3	0–0	0–0	0–0	25	140	70	0	N	N	100	100	0	100
12	E−	M	64	5	I	40	0–0	3–3	2–2	0–0	2–2	26	141	76	0	N	N	100	0	100	0
13	E−	M	65	8	I	50	0–0	3–3	3–3	0–0	2–2	28	100	56	0	N	N	100	0	100	0
14	E−	F	37	18	I	50	0–0	3–3	3–3	0–0	0–0	30	91	53	0	N	N	100	0	100	0
15	E−	M	68	8	I	30	0–0	3–3	0–0	0–0	0–0	30	131	79	1	N	N	100	0	100	0
16	E−	M	83	3	I	30	0–0	3–3	0–0	0–0	0–0	25	145	81	0	N	N	100	0	100	0
17	E−	M	48	13	I	101	0–0	3–3	0–0	0–0	0–0	30	144	82	0	N	N	100	0	100	0

*Id, patient’s code; G, group: presence (E+) or absence (E−) of embodiment of the co-experimenter’s arm (see misattribution column); S, sex; M, male; F, female; A, age; S, schooling: years of formal education; E, etiology; H, hemorrhage; I, ischemia; D, duration of the disease: number of days (d) between the onset of the disease and the first assessment; NE, neurological examination: contralesional motor (M), somatosensory (noxious and non-noxious stimuli; S), and visual half-field (V) neurological deficits (the two values refer to the upper and lower limb/visual quadrants, respectively); scores ranged from normal (0) to severe defects (3). A, anosognosia: unawareness of the motor (M), somatosensory (S), neurological deficits (the two values refer to the upper and lower limbs respectively); for the motor deficits, scores ranged from normal (0) to severe defects (3), whereas for the somatosensory deficits, scores ranged from normal (0) to severe defects (2). MMSE, mini mental state examination: cut off 24. Neglect: EP, extrapersonal; BIT-C, Behavioral Inattention Test – Conventional subtest, cut off 129; BIT-B, Behavioral Inattention Test – Behavioral subtest, cut off 67); P, personal; FLUFF test, cut off 2). Som, somatoparaphrenia: Y, yes; No, no; Aso, verbal asomatognosia: Y, yes; N, no; Mis, misattribution: one (arm) or two (arms) are present; Pt, patient; Co-ex, co-experimenter; numbers represent the % of times in which patient reaches that arm (eight trials)*.

**Figure 1:**
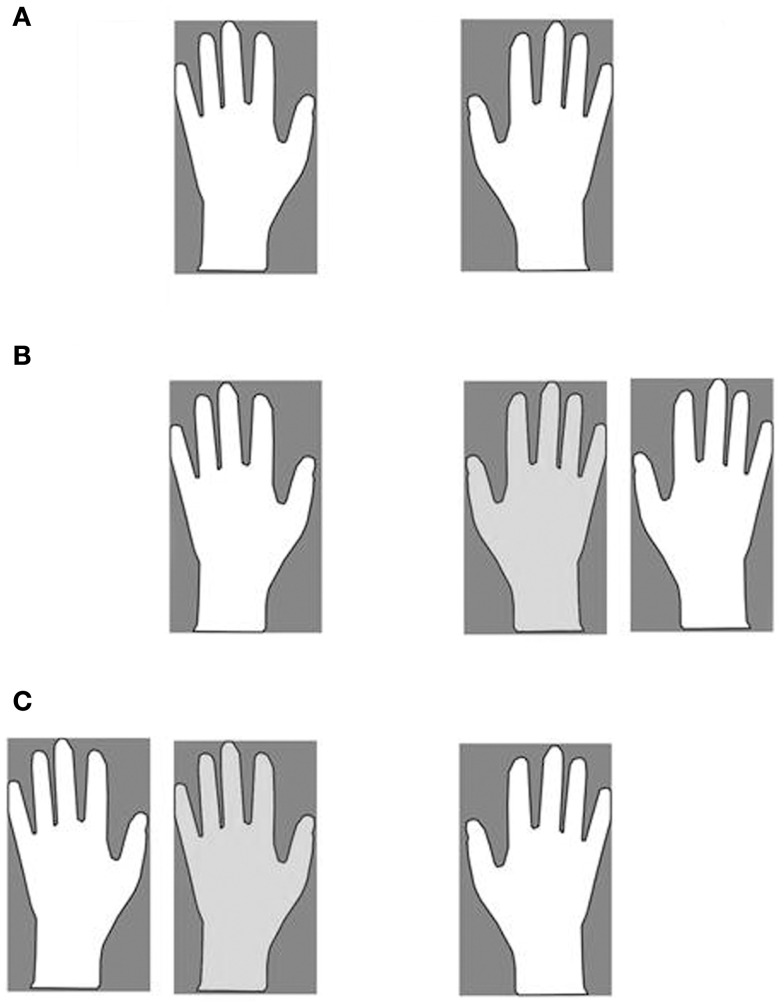
**The four experimental conditions**. Own **(A)** and Alien **(B,C)**. Participant’s hands (white), co-experimenter’s hand (light gray).

The misattribution of the co-experimenter arm was assessed in the following way: patients were requested to lie their arms on a table. A same-gender co-experimenter’s left (Figure 1C) or right (Figure 1B) arm was positioned on the same table, aligned with the patients’ trunk midline and internal with respect to the patients’ left (Figure 1C) or right (Figure 1B) arm. In one condition (Figure 1B), patients were asked to reach (eight trials) with their right, intact hand their own left hand and to name the color (eight trials) of the object positioned in front of their own left hand (in fact, three objects of different colors were placed in front of the own left and right hand, and the co-experimenter’s left hand). In another condition (Figure 1C), patients were asked to name the color (eight trials) of the object positioned in front of their own right hand. Respect to the right, all patients indicated (100%) the color of the object in front of their own hand. As regards the left, 11 patients consistently reached (90%) the co-experimenter’s hand (and named the color of the objects in front of the co-experimenter’s hand; hereinafter E+ group), whereas six reached (100%) their own hand (and named the color of the objects in front of their own hand; hereinafter E− group). It is worth noticing that patients correctly reached and (or) named their own hands when only their own arms were lying on the table (Figure 1A).

### Experimental task

Each participant sat in front of a table desk and a same-gender co-experimenter sat behind her/him. In the OWN condition, participants simply laid down their arms on the table (Figure 1A). In ALIEN conditions, the co-experimenter placed his/her left (or right) arm, on the table (by passing under the patient’s armpit), aligned with the participant trunk midline and positioned internally with respect to the participant’s left (or right) arm, respectively (Figures 1B,C). Hence, in the ALIEN condition, the co-experimenter’s hand was placed exactly where it was the participant’s hand in the OWN condition. A white sheet was draped over patient’s trunk, and arranged in order to prevent the direct vision of any body parts except hands. Noxious stimuli were administered by means of a homemade nociceptive stimulator with a cylindrical body in aluminum (length 20 cm, diameter 0.7 cm) and a retractable sharp tip in stainless steel able to apply fixed stimulus intensities (the exerted forces was about 500 mN). In the OWN condition, five stimuli for each participant’s hand dorsum were administered while in the ALIEN condition, the five stimuli were administered to each co-experimenter’s hand dorsum. The sequence was repeated twice (ABCD–DCBA order) and counterbalanced across participants. The total number of stimuli was forty. After each stimulation, participants were asked to rate the pain feelings evoked by the pinprick stimulators on a verbal rating scale (with 0 indicating “no pain,” and 5 indicating “maximal imaginable pain”). In order to control the effects of sensitization or fatigue, successive stimuli were applied in different spot of skin (some millimeters away). The mean ratings were employed to perform statistical analysis between and within groups.

## Results

**Figure 2:**
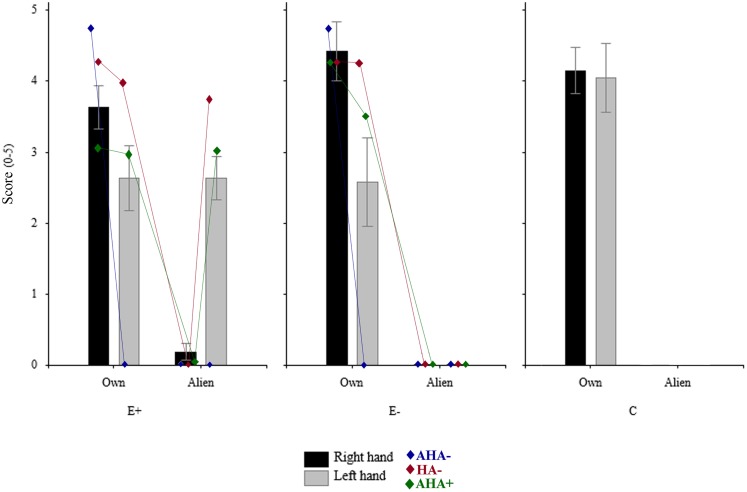
**Mean rating of the three groups (10 E+, 6 E–, and 10 C) in the four experimental conditions**. Rating of each subgroups obtained taking into account sensory deficits, and awareness of them (i.e., AHA−, hemianesthesia without anosognosia; HA−, no loss; AHA+, anosognosia for hemianesthesia) are also plotted. *Significant (*p* < 0.05); n.s., not significant (*p* > 0.05).

A repeated measures ANOVA on the mean score with OWNER (two levels: participant, co-experimenter) and HAND (left, right) as within-subjects factor, and GROUP (three levels: E+, E−, C) as between-subjects factor was performed (see Figure 2).

The main factor OWNER resulted to be significant [*F*(1,24) = 236.34, *p* < 0.00001)], namely participants gave a higher score when the stimulation was given on their own hands (mean = 3.579, SE = 0.201) respect to when it was administered to the co-experimenter’s hands (mean = 0.47, SE = 0.103). Also the OWNER × GROUP interaction was significant [*F*(2,24) = 15.26, *p* < 0.0001)], with the score given by the E+ group when the co-experimenter’s hands were stimulated (mean = 1.409, SE = 0.156) significantly (Duncan *post hoc* test *p* < 0.0001) higher respect to both E− (mean = 0, SE = 0) and C (mean = 0, SE = 0) groups. Crucially, the OWNER × GROUP × HAND interaction was significant.

As regards the right hand, each group gave a significant (Duncan *post hoc* test *p* < 0.00001) higher score when the stimulation was given on the own right hand (E+: mean = 3.64, SE = 0.306; E−: 4.416, SE = 0.414; C: 4.15, SE = 0.321) with respect to the co-experimenter’s right hand (E+: mean = 0.182, SE = 0.117; E−: 0, SE = 0; C: 0, SE = 0). Indeed, no significant between-groups differences were found either for the own right hand or for the co-experimenter’s right hand (Duncan *post hoc* test *p* > 0.05).

As regards the left hand, both E− and C groups gave a significant (Duncan *post hoc* test *p* < 0.0005) higher score when the stimulation was given on their own left hand (E−: mean = 2.583, SE = 0.623; C: mean = 4.05, SE = 0.483) than on the co-experimenter’s left hand (E−: mean = 0, SE = 0; C: mean = 0, SE = 0). One the contrary, the E+ group gave similar rating (Duncan *post hoc* test *p* > 0.05) when the stimulation was on the own left hand or on the co-experimenter’s left hand (own: mean = 2.636, SE = 0.46; alien: 2.636 = 0.305). Therefore for the left hand, significant between-groups differences were found (Duncan *post hoc* test *p* < 0.05). It is worth noting that in both E+ and E− groups the scores given to the own left hand was somehow lower than those given by the C group when their own left hand was stimulated (see above). This difference was mainly due to the presence in both groups of four patients who, being hemianesthesia, but not anosognosic, gave very low score to the capacity of the contralesional hand to perceive pain. Interestingly, the two E+ patients also gave low score to the co-experimenter’s, embodied, left hand.

## Discussion

With the present investigation, we aimed at examining the relationship between humans’ body ownership and the subjective experience of pain. We tested right brain-damaged patients who were convinced that the examiner’s left hand belonged to them. We asked whether (or not) such a (pathological) feeling of owning someone else’s hand can trigger pain perception each time the alien hand is stimulated with noxious stimuli. We predicted that if the alien hand is so deeply embodied in patients’ body representation, noxious stimuli might be referred to the patients’ body in absence of any concurrent stimulation of the own hand.

As expected, all participants correctly judged the delivering of noxious stimuli (i.e., gave scores significantly higher than zero) when their own right, but not the co-experimenter’s right was stimulated (the score did not differ between groups). Similarly, healthy subjects and patients without delusion of ownership gave scores higher than zero only when the stimulation was administered to their own left hand. Most importantly, and according to our prediction, patients who misattributed the alien hand to their body gave scores higher than zero, not only when their own left hand was stimulated, but also when noxious stimuli were delivered to the co-experimenter’s left hand. This result suggest that as long as an arm is subjectively perceived as part of one’s own body, painful stimuli delivered to an alien hand are experienced as if given to the own hand. In order to understand this puzzling phenomenon, it is crucial to examine the possible mechanisms underlying the pathological embodiment of the alien hand. The embodiment *per se* cannot be ascribed to the presence of personal neglect (i.e., inattention to the left side of the body): beyond the fact that the neuropsychological baseline assessment revealed its presence in only three patients (#6, 7#, and #9), the assessment of misattribution of the co-experimenter’s arm showed that E+ patients were perfectly able to reach their own left arm with their right (when the co-experimenter’s left was not present). Therefore the presence of personal neglect does not seem necessary to cause the delusion. Much more interesting is the fact that the delusion emerged only under some specific constraints. Firstly, it appeared when the co-experimenter’s left arm was placed parallel and internal, but not external, to the patient’s left arm. It is worth noticing that when the delusion emerged, E+ patients “saw” both arms, their own and the co-experimenter’s left, and often attributed their own to the co-experimenter (interestingly, these patients never displayed such a delusional beliefs about their own contralesional body part (Fotopoulou et al., 2011; Gandola et al., 2012), when only their own arms were lying on the table). Secondly, the delusion disappeared when the co-experimenter’s left arm was 180° -oriented (independently from its horizontal position with respect to the patient’s left arm). Finally, it disappeared also when the co-experimenter’s left arm was replaced with a rubber glove (independently from its position or horizontal orientation with respect to the patient’s left arm). We must emphasize that since we aimed at exclude a pure perceptual effect, the rubber glove was not realistic as in previous studies (Fotopoulou et al., 2008; Zeller et al., 2011) and participants clearly recognized it was non-human (see also below).

The interpretations of the rubber hand illusion effects on healthy participants may shed light on the above-mentioned constraints for the emergence of the delusion. There is a wide agreement in considering a bottom-up multisensory integration between vision and touch as a necessary condition for experiencing the illusion. However, for some authors (Armel and Ramachandran, 2003) this process is sufficient to generate the illusion. This, in turn, would predict the emergence of the illusion under a wide range of visual conditions as, for instance, when the rubber hand is in incongruent position with respect to the patient’s body or even when it is replaced by a non-human object. On the contrary, other authors (Tsakiris and Haggard, 2005) suggested that multisensory integration is not sufficient for the illusion to emerge because the on-line sensation must be necessarily compared to pre-existing body representations. This, in turn, would predict that the emergence of the illusion is constrained by these pre-existing representations of the body as the congruence in terms of position and identity.

In the present study, the conditions for the emergence of the pathological delusion of ownership are in line with the latter above-mentioned hypothesis (constraints imposed by the internal body representations). It is interesting to note that, despite in E+ patients the brain-damage has altered the normal body ownership (i.e., pathological embodiment), spared pre-existing representations of the body imposed limits on the type representation and its configuration. Hence, the congruence of the alien hand in terms of position and identity is necessary in order to accept an external object as belonging to one’s own body.

However, the fact that vision of someone else’s hand was sufficient to immediately produce the delusion differs from the rubber hand illusion in which repeated simultaneous stimulation of the fake and real hand is necessary for the illusion to emerge. It is interesting to note that a delusion of ownership due to the interaction between internal representations of the body with bottom-up unimodal (visual) stimuli has been reported also in healthy subjects (Slater et al., 2010). The authors showed that a first person perspective of a life-sized virtual human body that appears to substitute the participant’s own body was sufficient to generate a body transfer illusion. In other words, the authors demonstrated a delusion of ownership (i.e., a full body illusion) entirely due to visual capture mechanisms (i.e., without simultaneous synchronous tactile stimulations). Interestingly, first person perspective and level of skin realism were necessary in order the experience the illusion. This is in line with the fact that here the delusion disappeared when the co-experimenter’s left arm was 180° -oriented or with the rubber glove.

The second point we should discuss is why E+ patients reported pain feelings when noxious stimuli were delivered to the embodied arm. Some interesting hints come from examining the presence or absence of noxious/non-noxious deficits and awareness of noxious/non-noxious deficits in our sample of patients. Although unawareness of sensory deficits (AHA+ in Figure 2) seems to be more frequent in E+ (7 out of 11) than in E− (2 out of 6) patients, AHA+ seems to be not sufficient to explain the misattribution of painful perception. Indeed, the two AHA+ patients of the E− group did not experience pain when the left alien hand was stimulated. This seems to suggest that the subjective feeling of pain might be somehow related to an *a priori* embodiment of the alien hand. More importantly, E+ patients who acknowledged the sensory deficit on their own hand (AHA− in Figure 2) did not experience noxious stimuli on the left alien hand, coherently with their normal sensory awareness. This means that the alien embodied hand is subject to the similar sensory properties as one’s own hand. This is in line with the fact that when patients normally feel sensation on their left hand (HA− in Figure 2) or report to feel sensation on their left anesthetic hand due to the unawareness for the deficit (AHA+), the subjective feeling of pain delivered to the left alien hand is observed only in E+ patients.

Nonetheless, the crucial aspect related to the subjective feeling of pain when the co-experimenter’s hand is stimulated is the fact that stimuli must be seen. This is not trivial but, rather, consistent with the everyday experience that visual awareness of body parts can highly affects incoming tactile information. For instance, when an insect crawls on our skin, we do not experience any sensation if the stimulation is beyond the mechanical threshold. However, if we shift our sight toward the insect a vivid tactile experience can arise due to the interaction between localization and tactile noise. Other less anecdotic findings supports this idea. For example, right brain damages patients with partial sensory loss can report improved tactile sensation when they see the affected hand being touched (Halligan et al., 1997; Rorden et al., 1999). Moreover, in phantom limb patients, phantom pain can be ameliorated by superimposing the unaffected limb on the amputated one in a mirror (Ramachandran and Rogers-Ramachandran, 1996; MacLachlan et al., 2003) or by controlling a limb in virtual reality (Murray et al., 2007; Cole et al., 2009; Sato et al., 2010). Similarly, during the rubber hand illusion, potentially harmful or noxious stimuli approaching the rubber hand elicits the same brain activity (Ehrsson et al., 2007) and skin conductance response (Armel and Ramachandran, 2003) as when the healthy participant’s real hand is stimulated.

Tactile awareness, however, can be consciously reported even in situation in which the physical counterpart is absent (i.e., visual capture of touch). For instance, simply stroking a fake hand with a laser light can produce illusory thermal or tactile sensations in one’s own arm (Durgin et al., 2007). Similarly, in synesthetic individuals (i.e., people who experience a sensation in one modality when the stimulation is delivered in another sensory modality), the observation of another person being touched can be experienced as tactile stimulation on the equivalent part of one’s own body (Blakemore et al., 2005). Visual capture of touch has been interpreted in terms of a strong preference of the human’s brain to operate, in normal circumstances, under the principle of multisensory integration. This means that if input has a high certainty in one sensory modality, it can induce perceptual consequences in a different modality (Driver and Spence, 2000).

On this basis, it is possible to suggest that when E+ patients “saw” noxious stimuli delivered to a body parts that they subjectively perceive as own, they report painful feelings (as if stimuli were delivered to their own body). Note that the misattribution is not aspecific so to make them to experience all sort of stimuli delivered to whatsoever body part in the environment. On the contrary, it is circumscribed to the embodied alien arm and, as such, strictly related to the altered body representation. It is interesting to note that in our patients such a visual capture of touch might be independent from the ability to potentially perceive stimuli. Indeed, the two E+ patients with no sensory loss (HA−), attributed pain perception to the co-experimenter’s left hand despite they were able to feel tactile sensation on his/her left hand. In other words, being or not being able to feel does not affect the subjective feeling when the embodiment mechanism has induced the pathological body part attribution. Interestingly, the effect differed from the one reported during the rubber hand illusion (Capelari et al., 2009; Mohan et al., 2012) since here an altered feeling of body ownership can affect somatic sensation to a degree that it is possible to experience pain delivered to an alien hand in absence of any simultaneous stimulation of the own hand (in the rubber hand illusion the sensation is referred to the rubber hand while the own hand is receiving the stimulation). So far, only one study has reported similar findings (Aimola Davies and White, 2013). The authors administered a no-touch version of the rubber hand illusion (stimulation of the viewed prosthetic hand but no-touch of the participant’s hidden hand) to individuals with vision-touch synesthesia and healthy controls. Only synesthetics experienced the rubber hand illusion: the tactile sensation on their hand was referred to the prosthetic hand and their own hand resulted shifted toward the prosthetic hand.

The third point we should address is the possible neural basis of the delusion and of the illusory painful perception. It is crucial to emphasize that at the time of testing not all the MRI or CT scans were available and, hence, we were not able to map and analyze in depth the lesional pattern in the whole sample of patients. Nonetheless, an inspection of the existing scans suggested that putamen, dorsolateral prefrontal cortex, external capsule, parietal periventricular white matter and part of the insula might be more critically associated to the damages of the E+, rather than E−, group. Among the above-mentioned structures, some authors suggested that insular cortex might subserve pain processing (Coghill et al., 1994, 1999) and the subjective experience of one’s body (Karnath et al., 2005; Tsakiris et al., 2007). Nonetheless, damages to putamen and dorsolateral prefrontal cortex have been suggested to be crucial for the emergence of in such a delusion of ownership (Garbarini et al., 2013). Hence, these conclusions should be considered highly speculative and exhaustive anatomical analyses are needed.

The present data are in line with a study recently published by our group (Garbarini et al., 2013). In that paper, we demonstrated that the pathological embodiment of an alien hand can have objective consequences on the motor behavior of the intact hand. Indeed, in a bimanual task where subjects had to draw lines with the right hand and circles with the left, we found an ovalization of the lines when E+ patients observed an alien left hand drawing circles (the effect was similar to the one observed when healthy participants actually perform the task). It is interesting to note that, consistently with the above-mentioned constraints for the emergence of the delusion of ownership in E+ patients, coupling disappeared when the alien hand was arm was 180° -oriented. This effects indicate that the altered body ownership affects both motor awareness (despite usually aware of not being able to move, E+ patients, were convinced that their left hand was moving) and sense of agency (E+ patients ascribed the alien movements to themselves) by directly modulating action execution. These data suggested that the embodiment of someone else’s arm body can affect also internal motor programs.

Summarizing, we showed that the pathological delusion of owning an alien arm triggers pain perception when the alien hand is stimulated. We suggest that brain damages might have led these patients to assign ownership and visual (noxious) stimuli to an alien hand. Pre-existing (spared) models of the body distinguished between objects that may (or may not) be part of one’s own body on the basis of constraints (e.g., first person perspective, position with respect to the patient’s trunk midline, skin realism). In these conditions, if a noxious stimulus touches what is felt as looks like their own arm, this will be painful.

We must acknowledge a limit of the present investigation: we do not have any direct electrophysiological or neuroimaging data showing the activation of patients’ sensory processes. Hence, further studies are needed to answer this question. However, the phenomenon observed in E+ patients seems more likely to be explained in term of “perceiving” the stimulus rather than simply “reporting” what the patient see. Indeed, E+ patients aware that they could not feel any tactile stimulation on their own left hand (hemianesthesia without anosognosia), rated 0 noxious stimuli when both their own left and the co-experimenter’s (embodied) left hand was stimulated, whereas rated significantly higher than 0 noxious stimuli delivered to their own right hand. This means, at least, that the phenomenon is linked to sensory functions.

To conclude, further studies are needed to clarify the anatomo-physiological mechanisms responsible for both pathological attribution of other’s body part and the subjective experience of pain. Nonetheless, what clearly emerges from our data is that pain perception is not an all-or-none phenomenon, simply related to the direct bottom-up stimulation of nociceptors, but is intimately connected to the experience of body ownership that, in a top-down manner, may modulate self-consciousness and even personal identity (Merleau-Ponty, 1962; Edelman, 2004).

## Conflict of Interest Statement

The authors declare that the research was conducted in the absence of any commercial or financial relationships that could be construed as a potential conflict of interest.
